# Nanostructured Aerogels for Water Decontamination: Advances, Challenges, and Future Perspectives

**DOI:** 10.3390/nano15120901

**Published:** 2025-06-11

**Authors:** Alexa-Maria Croitoru, Adelina-Gabriela Niculescu, Alexandra Cătălina Bîrcă, Dan Eduard Mihaiescu, Marius Rădulescu, Alexandru Mihai Grumezescu

**Affiliations:** 1 Department of Science and Engineering of Oxide Materials and Nanomaterials, Faculty of Chemical Engineering and Biotechnologies, National University of Science and Technology Politehnica Bucharest, Gh. Polizu St. 1–7, 011061 Bucharest, Romania; alexa_maria.croitoru@upb.ro (A.-M.C.); adelina.niculescu@upb.ro (A.-G.N.); alexandra.birca@upb.ro (A.C.B.); 2 National Centre for Food Safety, National University of Science and Technology Politehnica Bucharest, Spl. Independentei 313, 060042 Bucharest, Romania; 3 Research Institute of the University of Bucharest—ICUB, University of Bucharest, 90–92 Panduri, 050663 Bucharest, Romania; 4 Department of Organic Chemistry, Faculty of Chemical Engineering and Biotechnologies, National University of Science and Technology Politehnica Bucharest, Gh. Polizu St. 1–7, 011061 Bucharest, Romania; dan.mihaiescu@upb.ro; 5 Department of Inorganic Chemistry, Physical Chemistry and Electrochemistry, National University of Science and Technology Politehnica Bucharest, 1-7 Polizu St., 011061 Bucharest, Romania; marius.radulescu@upb.ro

**Keywords:** aerogels, adsorption, pollutants, water treatment, sustainable production

## Abstract

Water contamination with toxic pollutants such as heavy metals, oil spills, organic and inorganic dyes, pesticides, etc., causes severe environmental and human health pollution. Aerogels have gained increasing attention in recent years as promising adsorbents due to their outstanding properties. This paper critically evaluates the recent advancements in aerogel-based materials, highlighting their challenges, limitations, and practical applications in large-scale experiments. The influence of key parameters such as adsorbent dosage, solution pH, ionic strength, and temperature is also discussed. Integrating nanotechnology and advanced manufacturing methods, a new generation of high-performance adsorbents with increased sorption capacity and reusability could be developed. Additionally, pilot studies and field trials are highlighted in this review, showing aerogels’ practical and real-world applications. Although various gaps in the production process that limit aerogel implementation in the market still exist, the research progress in the field shows that novel aerogels could be used in real wastewater treatment in the future. This review underscores the need for future research to develop advanced aerogel-based materials using green and sustainable synthesis methods that can lead to full-scale application.

## 1. Introduction

Although water is an essential resource found on Earth, less than 3% of the total amount is represented by freshwater, of which only 1% is available for human use [1,2]. Due to different factors, this limited resource is reaching a crisis point, becoming scarcer globally [3,4].

Key sources of water contamination include industrial emissions, domestic sewage, solid waste, pesticides and nitrogen fertilizers, petroleum spills, mining activities, etc. Consumption of polluted water can lead to serious human diseases and aquatic ecosystem degradation [5,6,7,8,9]. In this regard, developing effective wastewater treatment technologies is vital to remove persistent contaminants.

Current conventional techniques (e.g., membrane separation, ion exchange, coagulation, sedimentation, ozonation, reverse osmosis, etc.) have certain disadvantages because they often use expensive equipment and chemical reagents, they lack important technological tools, and they have a secondary negative impact on the environment [10,11]. Moreover, conventional wastewater treatment methods are unable to eliminate contaminants such as pesticides, organic dyes, pharmaceuticals, and heavy metals; thus, effective techniques (affordable, efficient, and environmentally friendly) are essential to be developed to successfully remove the persistent pollutants from the water [12,13,14].

Among the above-mentioned techniques, considerable attention has been paid to adsorption due to its lower cost and high efficiency [15]. In this regard, designing novel and efficient adsorbent materials that can eliminate these new and toxic contaminants is essential. In the last decade, numerous porous materials, including metal oxide, carbon-based materials, and metal–organic frameworks, have been explored for environmental applications, but most have disadvantages, such as high cost, complex fabrication methods, toxicity, and pollution. Among all solid materials, aerogels have been successfully used as novel adsorbent materials that can overcome these drawbacks and can be used for environmental applications to eliminate numerous types of pollutants contained in water [16,17,18].

Aerogels are ultra 3D-porous nanomaterials with a volume of air of 90–99%, being considered as one of the lightest solid materials nowadays. Aerogels are attractive due to their unique features, such as very low density, high thermal conductivity and good mechanical strength, large porous structure, large surface area, and high hydrophilicity [19,20,21,22].

Due to their particular properties, aerogels can be used in a wide range of applications, including drug delivery [23,24], biomedical applications [25,26,27,28], coatings [29,30], dye removal [31,32,33], oil spill removal [34,35,36], energy storage [37,38], electronics [39,40], sensing applications [41,42], etc. In addition, because of their superior adsorption capacity, they can be used for the removal of water contaminants, demonstrating high efficiency as adsorbents of dyes, organic solvents, and oils [43,44,45,46,47,48].

The first aerogel was fabricated in 1930 by Samuel Kistler using a silica-based material that eventually was used as an effective heat-insulating material. He used the supercritical drying method, forcing the liquid into a supercritical fluid state by increasing the temperature and pressure. Later, in the early 1940s, he and Monsanto designed silica aerogel products used in paints, napalm bombs, and silicone rubber [21,49]. Since the early 2000s, aerogels have shown high potential in advanced technological applications with increased research in developing new process techniques.

This review presents the application of aerogels in water decontamination in a comprehensive manner. Different types of aerogel-based materials (classified into silica, cellulose, chitosan, alginate, carbon hydrogels, hybrid, etc.) and their properties are also described. Moreover, this paper highlights the preparation techniques of aerogels and underlines the current challenges and limitations, encouraging future research and advancement in technology through an interdisciplinary perspective. Although recent advancements in aerogels for pollutant removal from aqueous samples have already been published in this domain, extensive research is still needed to optimize these materials’ properties and improve their adsorption for real-life applications.

## 2. Types of Aerogels Used in Water Decontamination

**Figure 1:**
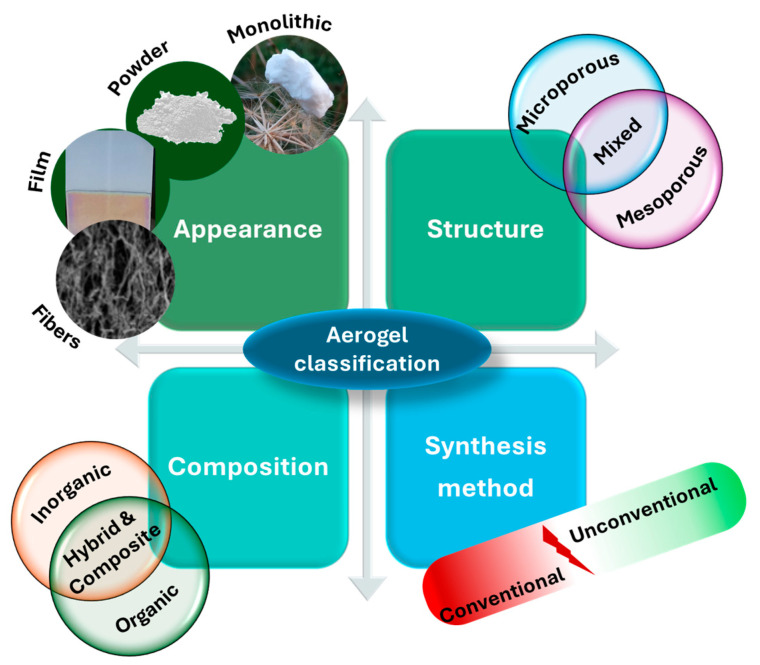
Classification of aerogels according to their appearance, synthesis methods, structure, and composition.

Aerogels can be obtained from different base materials such as chitosan, cellulose, alginate, carbon, graphene, silica, and many others. They are considered a distinctive class of materials with a unique 3D network of interconnected pores and specific characteristics, reflecting their potential for water-treatment applications [50,51,52]. They can be classified by the preparation method, appearance, structure, and composition (Figure 1).

**Figure 2:**
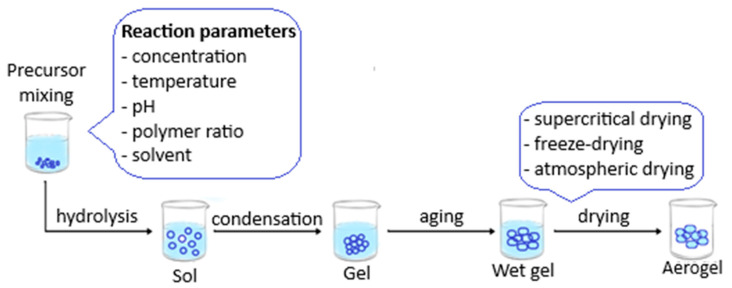
Schematic presentation of aerogel synthesis.

Because of their unique porous micro- or nanostructure, aerogels can adsorb various pollutants from aqueous samples. Moreover, their fabrication is considered simple, economical, facile, and safe. The most used process of producing aerogels is represented by the sol-gel method: the first step refers to the preparation of the solution and gelation; the process is followed by the elimination of the liquid without destroying the network structure; furthermore, drying is the last step and can be performed at ambient pressure, in supercritical conditions, or under vacuum [53,54,55]. The synthesis of aerogels is visually represented in Figure 2.

Furthermore, by combining/reinforcing them with different functional nanomaterials, the properties of aerogels can be improved (changes in pore structure, improved mechanical stability, larger specific surface area, more functional groups), and also their ability for adsorption of a wide range of toxic pollutants could significantly increase. Although the use of aerogels in water purification processes has been widely demonstrated in the literature, future research is still needed to enhance their overall efficiencies, as well as to lower their costs and improve sustainability [16,56].

### 2.1. Silica-Based Aerogels

Among all aerogels, silica aerogels have been widely studied and applied commercially and can be found in industrial quantities [55]. They are highly porous materials that consist of a crosslinked 3D network of SiO_2_ particles, and they stand out for their exceptional properties, including better structural control, highly porous structure, low density, high specific surface area, low thermal conductivity, and remarkable electrical conductivity [57,58].

Due to their outstanding characteristics can be applied in numerous areas, such as thermal insulation, humidity sensors, drug delivery materials, and adsorption catalysis [59,60,61,62]. Moreover, silica aerogels have received significant attention as novel adsorbents due to their excellent capacity for removing toxic pollutants from water.

Silica aerogels are usually obtained through a sol-gel process (consisting of gel preparation, aging of the gel, and drying process). During the first step, gelation occurs after mixing a silica solution with an organic solvent in the presence of a catalyst. During the aging step, the prepared gel is aged in its mother solution to strengthen the gel. The surface area, pore diameter, and volume are parameters that the aging solution concentration and aging time can influence. The drying process is the most important step and consists of removing the liquid from the gel pores. In this step, the removal of the liquid needs to be performed in a non-destructive way, and the gel structure needs to remain unchanged. Various kinds of drying have been proposed in this regard, such as high-temperature supercritical, low-temperature supercritical, ambient pressure, freeze-drying, microwave, and vacuum drying [63].

Although silica aerogels are designed using these three general steps, additional procedures are necessary to improve the final product properties. The low mechanical strength and fragile skeleton represent a major disadvantage of silica aerogels. Therefore, various techniques must be developed to strengthen the mechanical properties of aerogels. Thus, modifying silica aerogels with different materials can enhance characteristics such as mechanical strength, thermal and electrical conductivity, adsorption capacity, changes in pore structure, larger specific surface area, higher capacity for removing water contaminants, etc. [64,65].

Modified silica aerogels have shown improved characteristics for removing wastewater pollutants such as heavy metals, antibiotics, dyes, and other harmful substances [66,67]. For instance, Ghahremani et al. [68] synthesized a novel modified silica aerogel (NMSA) using the sol-gel method for removing Pb(II) ions from wastewater samples. This study added a small amount of Quince seed mucilage to the precursor solution to obtain a more effective adsorbent material. The NMSA demonstrated a 30% higher adsorption rate of Pb(II) ions than the raw silica aerogel. After the process was optimized, the results demonstrated a maximum Pb(II) removal efficiency of 75% at pH 4. Similarly, the excellent adsorption capacities for novel amidoxime-functionalized silica aerogels (AOSA-X) for eliminating heavy metal ions from the water were demonstrated. The results showed that AOSA3 exhibited a maximum adsorption performance of 598.05 mg⋅g^−1^ for Pb(II) and 534.10 mg⋅g^−1^ for Cu(II). Moreover, after five successive adsorption-desorption cycles, the adsorption rate of AOSA3 was higher than 86% [69].

Cheng et al. [70] designed a C8/threonine-modified mesoporous silica aerogel for the adsorption and desorption of both anionic (azophloxine) and cationic (methylene blue) dyes from wastewater. In the beginning, the sol-gel method was used to prepare the silica aerogel, and after that, through a silanization reaction, the surface was modified with n-octyl and threonine groups. The adsorption efficiency for both dyes was found to be 274.30 mg⋅g^−1^ (for methylene blue) and 152.43 mg⋅g^−1^ (for azophloxine), respectively. Additionally, the desorption capacity was more than 97%, demonstrating that C8/threonine-functionalized mesoporous silica aerogel is a promising adsorbent for both anionic and cationic dyes.

Reinforced silica aerogels can demonstrate improved characteristics for wastewater pollutants’ removal. Lamy-Mendez and collaborators investigated the removal capacities of carbon nanomaterials- methyltrimethoxysilane (MTMS)-based silica aerogel for various contaminants in aqueous solutions. The removal efficiency for MTMS-based aerogels was 170 mg⋅g^−1^ for toluene and 200 mg⋅g^−1^ for xylene. Adding carbon nanotubes increases removal efficiency by more than 71% for amoxicillin and 96% for naproxen. Thus, these novel materials demonstrate enhanced adsorption capacities, making them promising materials as industrial sorbents [31].

Another literature study demonstrated excellent adsorption performance for hydrophilic silica aerogel (HPSA) to remove Crystal violet (CV) dye from the aqueous solution. Compared to the previous literature report, the results showed a 96% removal of CV dyes after 30 min contact time and a pH of 7. The maximum adsorption capacity in equilibrium studies (Langmuir, Freundlich, Temkin, and D-R models) was 137.17 mg⋅g^−1^. These results showed great potential for HPSA aerogel as an efficient adsorbent for removing cationic dyes [71].

Developing a green, inexpensive, and effective adsorbent is needed to remove wastewater antibiotics. Thus, a polyvinyl alcohol (PVA)-assisted cellulose nanocrystals/SiO_2_ (CNCs/SiO_2_) was developed for effective removal of ciprofloxacin (CIP) from polluted water. With the initial concentration and temperature increase, the composite aerogel demonstrated a sorption capacity of Langmuir (qmax) for CIP of 163.34 mg·g^−1^. Adsorption of CIP by the PVA-assisted CNCs/SiO_2_ composite aerogel occurred due to mechanisms such as hydrogen bonding, π–π interaction, and hydrophobic and electrostatic interactions [72].

### 2.2. Carbon-Based Aerogels

In recent years, due to their exceptional properties, special attention has been given to carbon composite materials for the purification of different aqueous solutions. However, these materials also have some drawbacks, such as limited adsorption efficiency and low dispersion in water. To overcome these issues, the integration of carbon materials into aerogels can significantly improve the adsorption capacity for removing water contaminants [73,74].

Carbon-based aerogels have extraordinary adsorption performance due to their excellent characteristics, such as high surface area, low density, and large accessible pores. They have been successfully used for removing heavy metals, drugs, organic and inorganic dyes, pesticides, and other environmental pollutants [75].

A novel magnetic mesoporous carbon aerogel was designed using the citrate sol-gel method and examined for arsenic removal from water. The adsorption experiments revealed a maximum adsorption property of 56.2 mg·g^−1^ at pH 7.0. The results show that the magnetic mesoporous Fe_3_C/carbon aerogel can be used to remove toxic pollutants from water [76].

Among carbon aerogels, graphene and carbon nanotube aerogels exhibit comparable or better adsorption capabilities and are investigated for multiple applications, including wastewater treatment, chemical adsorbents, catalysts, etc. [74,77]. Gorgolis et al. [48] investigated the adsorption efficiency of different graphene aerogels for eliminating various toxic water pollutants. Two different drying methods were used for the synthesis of graphene aerogels: freeze-drying (FD) and ambient pressure drying (APD). The results revealed a superior adsorption capacity for APD Gas but a lower photocatalytic activity for most of the examined pollutants, compared to FD Gas, which showed a higher photocatalytic activity.

Although graphene aerogels exhibit great potential for removing various pollutants from water, graphene oxide (GO) aerogels are considered superior adsorbents because they exhibit better stability and performance and have excellent mechanical properties, high surface area, thermal resistance, and electrical conductivity [78]. The common methods to synthesize GO-based aerogels are self-assembly, chemical reduction, crosslinking, and sol-gel methods. The drying process used is usually freeze-drying or supercritical drying [54]. A recent scientific study evaluated the photocatalytic degradation of ibuprofen and sulfamethoxazole using reduced graphene oxide−TiO_2_/sodium alginate (RGOT/SA) aerogel. The results reveal that the obtained GO-based aerogel showed a removal capacity of over 99% of both contaminants within 45–90 min of exposure under UV-A light. Furthermore, at neutral pH, high photodegradation of Ibuprofen was observed, and at acidic to neutral pH, photodegradation of sulfamethoxazole occurred [79].

Mercury (Hg), considered one of the most toxic elements to human health, can bioaccumulate and cause serious adverse effects. Thus, developing effective adsorbents to remove Hg from water is necessary. In this regard, Bessa et al. [80] prepared a graphene oxide/polyethyleneimine (GOPEI) aerogel by the self-assembly method under acidic conditions (pH < 3) for Hg removal from real water. The results demonstrated a high removal efficiency of Hg from tap water (91%), river (90%), and sea (81%). Also, GOPEI aerogel still showed a high removal capacity after 3 successive sorption-desorption cycles.

The need for developing highly efficient composite materials for the photocatalytic removal of pollutants from water and air is increasing. Among most photocatalytic materials, 3D graphene-based gels are ideal candidates for increasing photocatalytic efficiency. For example, a 3D reduced graphene oxides/Mn_3_O_4_ (RGO/Mn_3_O_4_) aerogel was designed to remove antimonite (Sb(III)) and antimonate (Sb(V)). The experimental results show superior adsorption properties of 151.84 and 105.50 mg·g^−1^ toward Sb (III) and Sb (V), respectively. Due to the excellent adsorption capacity of 3D RGO/Mn_3_O_4_ aerogel in a wide range of pH, changing toxic heavy metal ions into non-toxic ones is essential for the photocatalytic removal of these ions [81].

### 2.3. Biopolymer-Based Aerogels

It is well known that aerogels can also be designed using organic and inorganic natural materials. Specifically, polymer-based aerogels have excellent properties such as high surface area, the ability to decrease their hydrophilicity, extremely low density and thermal conductivity, and enhanced adsorption performance for the removal of various pollutants in water [82]. Compared to synthetic polymers, natural polymers (e.g., chitosan, alginate, proteins, cellulose, etc.) represent an eco-friendly and renewable alternative for developing biopolymeric-based aerogels. Moreover, aerogel-based biopolymers possess greater adsorption properties and are more efficient in removing dye and separating oil and water. These innovative materials are highly attractive because they can be obtained from natural sources, and they can be reused after several cycles to maintain biodegradability. They also represent a sustainable option because they have a more controllable structure, better physico-chemical properties, and are non-toxic and biocompatible. Due to their abundance and natural availability, they can be obtained on an industrial scale, making them suited to fabricate unique aerogels with desired properties [83].

Due to their abundance and extraordinary properties, cellulose and chitosan are the most widely used biopolymers employed for aerogel production. Among water pollutants, pharmaceuticals (such as antibiotics, analgesics, anti-inflammatories, etc.) are considered extremely toxic to human health and the environment due to their side effects. A novel functionalized cellulose aerogel was fabricated by Lv et al. [84] using cellulose nanocrystalline (CNC) modified with polyvinylamine (PVAm) and reduced graphene oxide (rGO) for the adsorption of diclofenac sodium (DCF). The results showed a maximum adsorption capacity of 605.87 mg g^−1^, which was 53 times bigger than that of bare CNC aerogel (11.45 mg g^−1^). Moreover, DCF was easily desorbed with a solution of 0.1 M of NaOH, and the absorbent could be reused four times.

As mentioned above, biopolymer-based aerogels are excellent adsorbents for dye removal. Although chitosan aerogels have been reported to be effective in dye removal, they still have some drawbacks in practical applications. Thus, they need to be functionalized with different materials for better results. In this regard, a chitosan/zeolite composite aerogel was synthesized and evaluated for its adsorption of both anionic (indigo carmine) and cationic (methylene blue) dyes. The CS–ZX aerogel demonstrated good adsorption ability for both dyes, with a maximum uptake capacity of 221 mg g^−1^ for indigo carmine and 108 mg g^−1^ for methylene blue, respectively. Moreover, the composite aerogel could be reused for three adsorption-desorption cycles [85]. Lyu et al. [43] developed a 3D polyaniline/cellulose nanofiber aerogel (PCNFA) using a two-step method for the removal of Acid Red G (ARG) and Methyl Blue (MB) dyes. The batch adsorption studies revealed a maximum adsorption capacity of 600.7 mg g^−1^ for ARG and 1369.1 mg g^−1^ for MB dye. Compared to the adsorption studies for pure cellulose nanofibers (87.7 mg g^−1^ for ARG and 496.4 mg g^−1^ for MB, respectively) and polyaniline nanofibers (211.5 mg g^−1^ for ARG and 70.13 mg g^−1^ for MB, respectively), PCNFA aerogel demonstrated superior adsorption performances. Regeneration and reusability studies using the peroxy disulfate (PDS) method showed that ARG and MB dyes were successfully desorbed from the aerogel.

The adsorption efficiency of heavy metal ions, organic solvents, and oils from aqueous solutions was evaluated using polyurea-crosslinked Ca-alginate (X-Ca-alginate) aerogel beads. The maximum adsorption capacity of the X-Ca-alginate aerogel beads for Pb (II) was 29 mg g^−1^. The maximum volume uptake for organic solvents was 6 mL/g^−1^, and the absorption of oils (diesel, mineral, and pump oils) was 4.9, 6.1, and 4.9 mL/g^−1^, respectively. Moreover, the aerogel beads could be reused at least two times after treatment with a Na_2_EDTA solution. These results indicate that X-Ca-alginate aerogels can be used as effective adsorbents for removing a wide range of contaminants from water [86].

### 2.4. Hybrid and Composite Aerogels

In order to enhance the mechanical and thermal properties and to strengthen the nanostructure of polymer aerogels, functionalization with metal oxides such as TiO_2_, ZnO, Al_2_O_3,_ Cr_2_O_3_, etc., represents a good solution to prepare hybrid aerogels [87]. Due to their extraordinary properties, such as high adsorption kinetics, great regeneration and reusability, large pore volumes, and improved stability, hybrid aerogels are very effective in the removal of heavy metal ions from aqueous solutions. Although they have some drawbacks (time-consuming and long fabrication process), hybrid aerogels have demonstrated better performance in water purification applications [82]. These metal oxides are also used as photocatalysts, either single or combined with GO or CNT, to remove various water contaminants [88,89].

Nawaz et al. [90] examined the photocatalytic degradation of microcystin-LR in aqueous solution using GO-TiO_2_/sodium alginate and rGO-TiO_2_/sodium alginate aerogels. The conducted tests revealed better results for rGO-TiO_2_/sodium alginate aerogel that completely degrades microcystin-LR. Also, the highest adsorption performance (<90%) was obtained by rGO-TiO_2_/sodium alginate aerogel. The recyclability, high stability, and degradation efficiency make the rGO-TiO_2_/sodium alginate aerogel a more efficient photocatalytic system. Similarly, the TiO_2_-sodium alginate composite aerogels (SAT) were fabricated using a “green” approach and examined for oil-water separation. The SAT aerogel exhibited excellent oil/water separation capacity (up to 99.7%) and excellent photocatalytic activity for methyl orange (MO) degradation (>85% after 6 cycles) [91].

Yao et al. [92] prepared copper ferrite/reduced graphene oxide (CF/rGO) aerogel as a highly efficient catalyst for the removal of rhodamine B dye. The kinetic results for the degradation of RhB using CF/rGO aerogel demonstrated a maximum removal efficiency of 95.7% in just 1.0 min. The reusability studies showed a slight degradation of the CF/rGO aerogel after 5.0 min, but the removal efficiency was 87.4% after 5 cycles, thus demonstrating good stability overall. Novel 3D graphene/δ-MnO_2_ aerogels were synthesized and used as adsorbents to remove heavy metal ions from aqueous solutions. The 3D aerogels exhibited a maximum adsorption capacity of 643.62 mg g^−1^ for Pb^2+^, 250.31 mg g^−1^ for Cd^2+^, and 228.46 mg g^−1^ for Cu^2+^, respectively. Furthermore, regeneration and reusability studies were conducted, and the results demonstrated that aerogels can be reused for more than eight cycles while maintaining their original shape. These excellent results make these hybrid aerogels suitable for heavy metal ion removal [93].

In some cases, adsorbents such as GO can have limited applications in water decontamination due to their hard separation from water. Thus, the use of magnetic separation has become more attractive because the separation is more easily achieved by using an external magnetic field. In this regard, Fe_3_O_4_ magnetic NPs demonstrated strong magnetic properties and great biocompatibility, chemical stability, low cost, and easy preparation [81,94].

Arabkhani and collaborators investigated the adsorption process of a novel 3D magnetic bacterial cellulose nanofiber/GO aerogel (MBCNF/GOPA) for malachite green removal. The adsorption efficiency was measured using different pH solutions ranging from 2 to 12, and the results revealed that the adsorption efficiency increased gradually from 54% to 90%. The Langmuir isotherm model showed a maximum adsorption of 270.27 mg g^−1^. In the reusability tests, after seven cycles of adsorption–desorption in acetic acid/methanol solution, the adsorption efficiency was 62.7%. Moreover, MBCNF/GOPA aerogels can easily be collected from water with a small magnet [95]. Similarly, the efficiency in removing cation and anion dyes of a novel magnetic carboxymethyl chitosan (Fe_3_O_4_@PDA/CMC) aerogel was examined. The obtained aerogel showed a maximum adsorption capacity of 289.6 and 275.2 mg g^−1^ for the two cationic dyes (Methylene blue and Crystal violet) and of 82.07 and 92.35 mg g^−1^ for the two anionic dyes (Methyl orange and Congo red. Furthermore, the Fe_3_O_4_@PDA/CMC aerogel reveals superior regeneration properties after seven adsorption-desorption cycles and can be reused using a small magnet after every cycle [96]. Li et al. [67] synthesized a novel magnetic carbon aerogel (MCA) containing Fe_3_O_4_ nanoparticles and sodium alginate (SA) as the main carbon source for the removal of Cd(II) ions from water. The authors reported that their aerogel possessed an outstanding adsorption capacity of 143.88 mg g^−1^ for aqueous Cd(II). Moreover, Fe_3_O_4_ nanoparticles can easily be recovered from water using only a magnet. Table 1 presents the applications of aerogels6 in water decontamination.

## 3. Mechanisms of Pollutant Removal Using Aerogels

The presence of pollutants in water—even in small quantities—greatly impacts all living organisms and the environment. Thus, the interest in using efficient adsorbents is increasing to reduce the impact of pollutants on the environment. Aerogels have recently been investigated as adsorbents for removing pollutants from aqueous solutions. Research literature reports have shown that adsorption is a widely used technique for water decontamination, removing heavy metals, dyes, pesticides, pharmaceuticals, organic pollutants, etc. As adsorbents, aerogels have extraordinary characteristics, such as high specific surface area, low density, thermal conductivity, stability, and tunable pore size and shape [82].

**Figure 3:**
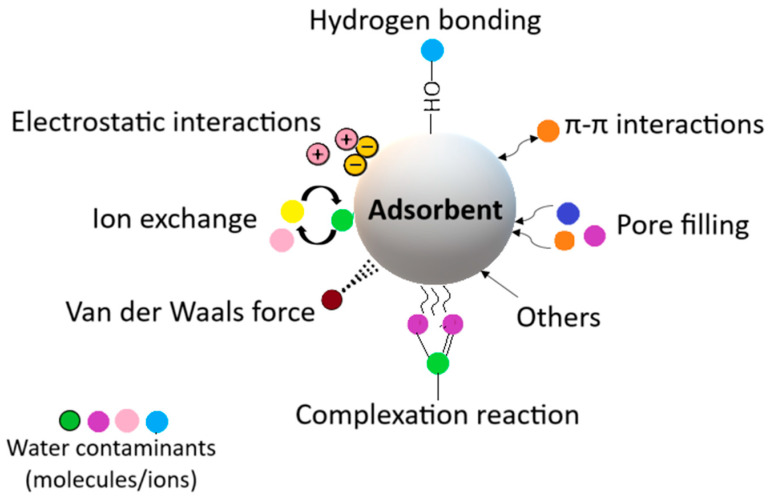
Mechanisms of the adsorption process.

These important parameters influence the adsorption capabilities, and by adjusting them through functionalization, the adsorption capacity of aerogels can be enhanced and used in desired applications. Thus, different organic, inorganic, and hybrid aerogels have been developed with better pore size distribution and chemical composition to satisfy the needs of adsorption applications. The adsorption process is based on ion exchange, and it usually follows the Langmuir isotherm and pseudo-second-order kinetics [97]. The adsorption mechanism is shown in Figure 3.

Adsorption isotherms show the interaction between adsorbates and adsorbents at constant temperatures. The Langmuir isotherm model indicates that maximum adsorption takes place when a saturated monolayer of adsorbate molecules is present on a homogenous surface. The Freundlich isotherm model is particularly effective for adsorption on heterogeneous surfaces, indicating that different binding sites have various adsorption affinities and associated capacities. By analyzing these adsorption isotherms, researchers can better understand the adsorption mechanism, performance, and efficiency of different adsorbents under diverse conditions [98]. With regards to kinetics, pseudo-second-order is the superior model used to describe adsorption kinetics. Kinetic studies provide information about the rate at which adsorbates are removed from the aqueous solution by examining factors such as temperature, adsorbate concentration, and contact time [99,100].

The solution pH is also important in adsorption; it can affect the adsorbent surface, ionization degree, solution chemistry, and the removal efficiency of pollutants [101]. Chen et al. [102] studied the adsorption performance of a novel rGO aerogel for removing Cr(VI) anions from water at different pH levels (from 1 to 8). The obtained results revealed the maximum adsorption performance of 361 mg g^−1^ at pH 2. The adsorption capacity decreased with the increase in the pH value of the solution. Cheng et al. [103] also demonstrated the adsorption efficiency of 3D-aminosilyated nanocellulose aerogels (APTMS-modified TO-NFC) for removing heavy metal ions by changing the pH value of the solution. The designed aerogel shows good adsorption capacity of 99.0, 124.5, and 242.1 mg g^−1^ for Cu^2+^, Cd^2+^, and Hg^2+^, respectively. These maximum adsorption capacities were achieved at pH values between 3 and 7.

Temperature is another important factor that influences adsorption efficiency. With the increase in temperature, the reaction rate increases in endothermic reactions and decreases in exothermic reactions [104].

Wang et al. [105] prepared a calcium Alg-EDTA aerogel for removing heavy metal ions from aqueous solutions. Experimental results showed a maximum adsorption efficiency of more than 85% for Cd^2+^, Pb^2+^, Cu^2+^, Cr^3+^, and Co^2+^ at pH values between 4 and 6.5. Furthermore, oil/water separation can be controlled by changing temperature (a lower temperature makes the materials hydrophilic, and a higher temperature makes the materials hydrophobic). For example, a research study demonstrated that by adjusting temperature and pH parameters, silica aerogels can change their wettability and act as promising oil/water separation materials. At a temperature below 34.73 °C and pH > 7, silica aerogel can adsorb water, but a temperature above 34.73 °C and pH < 7 makes the silica aerogel adsorb oil [106].

Another key parameter to be considered in the adsorption processes is the ionic strength of an aqueous solution. Usually, contaminants like heavy metals, dyes, and pharmaceutical agents contain different electrolyte ions that can influence the adsorption processes by changing the parameters of adsorbents and adsorbates [107]. A scientific study evaluated the effect of ionic strength on Cu(II) adsorption using a 3D-porous aerogel based on citrus peel (CP), chitosan (CS), and bentonite (BT). The adsorption experiments were conducted in the presence of a Cu(II) solution containing 0.1 M NaNO_3_. The results demonstrated that the removal rate of Cu(II) is independent of the liquid’s ionic strength, showing the adaptability of the synthesized aerogel in different water matrices [108]. Another recent study investigated the adsorption process of Cr(VI) on biochar/reduced graphene oxide aerogel (BC/rGA) in different environmental conditions. The experiments were realized in a Cr(VI) solution containing K_2_Cr_2_O_7_ dissolved in 0.01 M NaCl (pH 2). The results showed that with the increase in Cr(VI) concentration, the adsorption process of Cr(VI) on BA/rGA aerogel decreased. Thus, the ionic strength and pH parameters affected the adsorption capacity [109].

### 3.1. Photocatalytic Degradation

In the past few years, the design of innovative adsorbents with improved characteristics such as longer lifespan, low cost, ability to regenerate and reuse, and superior adsorption properties for removing a wider range of pollutants from water (including microplastics and per- and poly-fluoroalkyl substances) has become extremely important. Moreover, using these adsorbents with advanced water treatment techniques, such as photocatalytic degradation, oxidation processes, and biodegradation, can lead to improved performance in decontamination [110,111].

**Figure 4:**
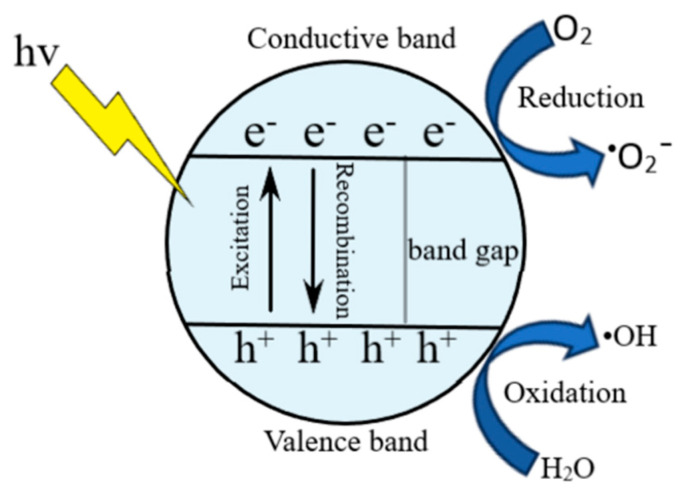
General mechanisms of the photocatalytic degradation.

Photocatalytic degradation is a technique that complements the adsorption process, thus being more effective in removing hazardous organic pollutants (Figure 4). Moreover, new photocatalytic platforms are being developed to successfully degrade numerous contaminants. Among these materials, nanostructured metal oxides such as TiO_2_, g-C_3_N_4_, and ZnO demonstrated their efficiency in removing organic pollutants using adsorption and photocatalytic degradation. More importantly, many studies have proved the extraordinary advantages of aerogels in adsorption and photocatalysis due to their above-mentioned properties. Thus, combining aerogels with photocatalysts can improve their properties and adsorption processes and expand their utilization range [112].

Gallegos-Cerda et al. [113] developed a novel photocatalytic aerogel based on cellulose, carbon nanotubes, and TiO_2_ nanoparticles to degrade organic dyes (rhodamine B and methylene blue). The degradation process was evaluated by UV–Vis spectroscopy, and the results showed that photocatalytic removal was more than 97% after 110 min of UV irradiation. Recently, Liu et al. [114] studied the photocatalytic degradation of Rhodamine B (RhB) using 3D-cellulose/graphene/carbon nitride (CNF/rGO/g-C_3_N_4_) aerogels. The photocatalytic performance was investigated under 300 W Xe lamp visible light, and the results demonstrated a degradation rate of RhB of 87.3%. Furthermore, the CNF/rGO/g-C_3_N_4_ aerogels exhibited excellent recycling activity and can be successfully reused.

Photocatalysis is also used to degrade pollutant antibiotics. For example, Yang et al. [115] prepared a novel agar/carbon dots/graphitic carbon nitride (Agar/CDs/g-C_3_N_4_) aerogel for photodegradation of amoxicillin (AMX) under visible light irradiation. After 45 min of illumination, Agar/CDs/g-C_3_N_4_ aerogel completely degraded AMX due to the material’s interconnected porous structure and wider visible light range. Photodegradation experiments showed enhanced photocatalytic activity for the prepared aerogel.

Recently, there has been a growing interest in obtaining novel treatment methods for combating antibiotic-resistant bacteria. A new approach for microbial disinfection is based on photocatalysis, which is based on generating reactive oxygen species (ROS) that cause bacterial cell death through oxidative stress. In the past few years, important semiconductor materials have been used in photocatalysis processes. Among them, TiO_2_ nanoparticles demonstrated efficiency as great antibacterial photocatalysts. Moreover, SiO_2_/TiO_2_ aerogels are excellent materials with improved properties that act as excellent adsorbents and photocatalysts with antibacterial activity under visible light [116].

Tiryaki and collaborators studied the photocatalytic activities and phototoxic effects of SiO_2_/TiO_2_/Ag@Au nanostars for the degradation of Rhodamine B (RhB) organic dye and against *Escherichia coli*, respectively. Photodegradation studies revealed enhanced photocatalytic efficiency under visible light irradiation due to SiO_2_/TiO_2_/Ag@Au aerogel’s improved properties. The phototoxicity against *E. coli* revealed that the prepared aerogel killed all the bacteria after 3 h of exposure to visible light [117].

For an at-a-glance comparison among the presented aerogels for photocatalytic degradation, Table 2 summarizes key information.

**Table 2:** Applications of aerogels in photocatalytic degradation.

Type of Aerogel	Pollutant	Removal Rate (%)	Time (min)	Type of Light	Reusability
Cellulose/CNT/TiO_2_	Rhodamine B Methylene blue	>97	110	UV light	N/A
3D-cellulose/graphene/carbon nitride	Rhodamine B	87.3	N/A	Visible light	100 cycles
Agar/carbon dots/graphitic carbon nitride	Amoxicillin	100	45	Visible light	6 cycles

### 3.2. Oil–Water Separation and Hydrophobic Aerogels

Water pollution caused by frequent oil spill accidents is becoming an urgent problem that needs to be solved due to its bad effects on the environment and ecosystem. Thus, it is imperative to design effective oil–water separating materials with high absorption capacity and low cost. Hydrophobic aerogels have attracted researchers’ attention due to their extraordinary properties, demonstrating higher oil absorption capacity because of their hydrophobic and oleophilic nature. Among them, silica aerogels, cellulose-based materials, and carbon-based aerogels have been studied as great candidates for oil spill absorption. However, the hydrophobic structure of these aerogels can be obtained by removing oxygen-containing functional groups, usually by carbonization, chemical treatment, or the use of cross-linkers. The water contact angle (θ) is measured to determine the hydrophobic properties of aerogels. Thus, a hydrophobic hydrogel has a water contact angle of θ ˃ 90° and repels water, but if the θ ˂ 90°, the hydrogel is hydrophilic and absorbs the water droplets [118,119,120].

**Figure 5:**
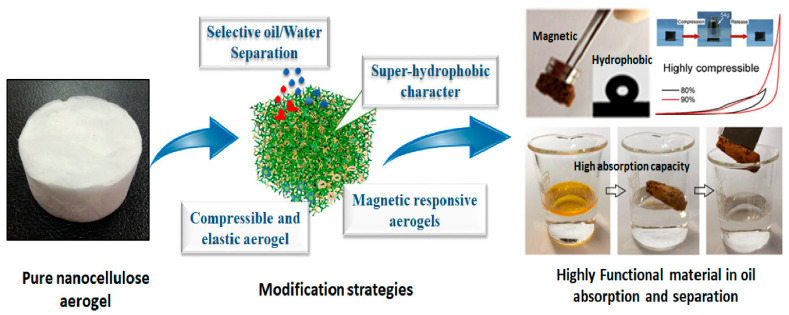
Illustration of modification processes for nanocellulose aerogels for oil absorption and separation. Reprinted from an open-access source [121].

Despite the extraordinary advantages of aerogels in water purification, the large specific surface area and high porosity make them highly hydrophilic, leading to poor oil/water selectivity and low separation efficacy. Thus, several modification strategies, such as physical or chemical methods, have been applied to develop ideal, cost-effective absorbents with high oil absorption capacity and improved oil/water selectivity (Figure 5) [121,122].

**Figure 6:**
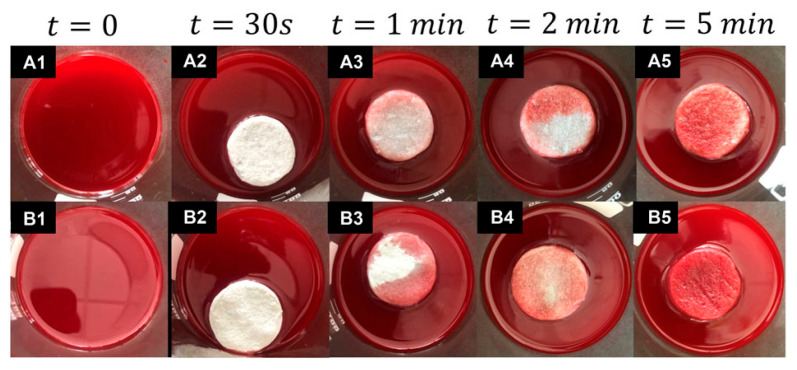
Mineral oil absorption for WOPP (**A1**–**A5**) and WOPW (**B1**–**B5**). Reprinted from an open-access source [124].

Sun et al. [123] prepared hydrophobic/oleophilic cellulose/SiO_2_ aerogels and tested their surface wettability and absorption capacity for oils and organic solvents. The experiments indicated excellent hydrophobicity (contact angle of 143°) and oleophilicity (contact angle down to 0°). Furthermore, the absorption experiments demonstrated that cellulose/SiO_2_ aerogels can absorb oils and organic solvents, increasing their mass by 16∼40 times. These results demonstrate that the obtained aerogels represent a viable oil/water separation option. Similarly, Montero et al. [124] synthesized hydrophobic wastepaper cellulose-candelilla wax aerogel (WOPW) and wastepaper cellulose-polycaprolactone (WOPP) aerogel and studied their efficacy in adsorbing organic solvents and oils. The contact angle tests revealed a value of more than 120° for both materials, which indicates a highly hydrophobic nature. The absorption tests were also realized, and the results showed a maximum adsorption capacity of 6.1 g/g in 5 min for WOPW aerogel and 4.88 g/g for WOPP aerogel (Figure 6). Remarkably, the WOPW aerogel can absorb mineral oil seven times its weight, showing its exceptional performance.

Hydrophobic carbon fiber aerogels (CFA) from black locust (BL), hybrid polar (HP), and chestnut shell (CS) were designed, and their effect on removing petroleum-based pollutants and vegetable oil was determined. The test results indicated a water contact angle of 141° for CFA from BL, 136° for CFA from HP, and 124° for CFA from CS, demonstrating the aerogels’ hydrophobicity. The oil sorption capacity was also measured, and the CFA aerogels from HP showed a maximum absorption capacity of 81.6 g/g and 76.0 g/g for crude oil and diesel oil, almost twice as big as the absorption capacity of CFAs from CS or BL. For sunflower oil, the sorption tests revealed the highest value of 81.8 g/g for the CFA from BL [125].

### 3.3. Electrochemical and Membrane Applications

Water scarcity and contamination represent a major global challenge; hence, developing cost-effective and efficient wastewater treatment methods to obtain clean drinking water is essential. Moreover, in order to increase the availability of fresh water, the desalination of brackish water is also important, as studied in research reports. One of the viable water desalination technologies with great potential is capacitive deionization (CDI) [126]. This method represents a great alternative to thermal methods, electrodialysis, and reverse osmosis because of its low energy consumption and low cost of production [127]. The principle of CDI systems depends on the type of electrodes used and their electrochemical properties. Thus, more recently, different porous carbon aerogel materials were studied as effective CDI systems due to their high ion adsorption/desorption capacity, large specific surface area, good electrical conductivity, tunable structure, and low-cost fabrication. For example, Beke et al. [128] prepared a carbon aerogel polypyrrole (CA-PPy) composite to desalinate NaCl solution using the CDI technique. The electrochemical tests were performed in a 1 M KCl electrolyte solution, and the results showed a specific capacitance of the CA-PPy composite of 360.1 F/g. The CDI desalination studies demonstrated a salt adsorption capacity ranging from 10.10 mg/g in 300 mg/L NaCl solution to 15.7 mg/g in 800 mg/L NaCl solution. The material also showed good electrochemical stability after nine cycles of ion adsorption/desorption tests. Similarly, Zhang et al. [129] reported the salt adsorption effect of carbon aerogel (CA) electrodes for Cu^2+^ in CuSO_4_ solution using a CDI desalination experiment. The results revealed the highest adsorption efficiency of 29.7 mg/g for the 1030 CA sample, due to its large amount of micropores in the structure. These results show that CAs can be used in heavy metal ion treatment.

A recent study evaluated the adsorption properties of sugarcane bagasse/ activated cellulose carbon aerogel-sodium alginate (SB-ACCA-SA) and SB-ACCA-SA-CNT electrodes through the desalination process using a CDI system. The salt adsorption capacity for SB-ACCA-SA was 17.87 mg g^−1^, but after CNT was added, it increased to 31.40 mg g^−1^. Moreover, even after 60 cycles, the desalination process using the SB-ACCA-SA-CNT electrode was stable [130,131].

Another important process used in wastewater treatment is membrane technology due to its advantages, such as low cost, low use of chemicals, environmentally friendly, and high productivity. Usually, membrane technology includes ultrafiltration, microfiltration, nanofiltration, electrodialysis, and reverse osmosis. The membrane process works by preventing smaller substances from a liquid from passing through the membrane and allowing only clean water. Although membrane filtration techniques have received significant attention in treating wastewater, there are some parameters that affect the performance and quality of the membrane, such as pore size, type of material, membrane degradation, etc. Therefore, more efficient and environmentally friendly approaches for improving these factors need to be developed and applied for advanced water treatment [132,133].

In this regard, hybrid membrane processes are a novel approach that has a better performance than any standalone membrane technology. These membrane processes are obtained by combining one or more membrane techniques with other membrane processes (such as ion exchange, adsorption, coagulation, humidification, etc.) for better separation efficiencies and cost [134,135]. For example, Ashar et al. [136] studied the photocatalytic degradation of reactive Black 5 (RB5) dye under artificial sunlight by using undoped and a series of Fe^3+^ doped ZnO in photocatalytic membrane reactors (PMRs). The RB5 dye degradation was monitored by UV/vis spectroscopy, and the results demonstrated a maximum degradation rate of 88.89% for ZnO/PMR and 98.34% for Fe^3+^@ZnO PMR in 180 min. Furthermore, after eight batches, the photocatalytic efficiency of Fe^3+^@ZnO PMR gradually decreased.

The development of environmentally friendly novel processes for metal ion removal from contaminated waters is very important for sustainable development. Complexation–ultrafiltration (CP–UF) could be a promising technique for heavy metal removal. A recent study investigated the use of polyacrylic acid sodium (PAAS) as a complexation agent for the removal of cadmium (II) ions. Two kinds of rotating disks with different rotating speeds were used. Cd (II) rejection reached 99.7% when the rotating speed was lower than 2370 and 1320 rpm for both disks. However, a higher rotating speed led to a decrease in Cd (II) ions. Also, the PAAS can be regenerated by shear-induced dissociation and ultrafiltration [137].

## 4. Current Challenges and Limitations in Aerogel Applications

In the last decade, significant progress has been made in developing advanced adsorbents with improved adsorption capacities and specificities. Nevertheless, several challenges and limitations remain. One of the major drawbacks in synthesizing adsorbents such as graphene-based materials, magnetic nanoparticles, metal-oxides, and metal-organic frameworks is the high production cost, leading to difficulties in large-scale production and commercialization. Thus, it is crucial to develop less expensive adsorbents by using environmentally friendly and innovative synthesis techniques [111,138,139]. Using safe and green chemicals and less complicated drying stages would boost the large-scale preparation of aerogels [140]. Moreover, aerogels are prized for their mechanical properties for various industrial applications. Modifying aerogel structures with different materials can improve mechanical strength and adsorption performance [141]. Tarashi et al. [142] developed a κ-carrageenan/polyacrylamide /GO aerogel with superior mechanical properties for the absorption of water pollutants. The mechanical properties were enhanced by using a double network strategy. The mechanical and adsorption performances were investigated, and the results demonstrated excellent compressive strength (25.902 MPa) and maximum adsorption capacities of 105.18 mg/g for methylene blue and 42.95 mg/g for Congo red. Silica aerogels are known for their poor mechanical properties and low strength. To increase these properties, various strategies were developed, including functionalization with organic polymers, the selection of silane precursors, and the incorporation of organic nanofibers into silica aerogels. In this sense, a recent study investigated the mechanical properties of silica aerogels reinforced with embedded fibers. A mixture of aramid fibers and nanofibers was used to increase the strength of silica aerogels. The samples showed improved mechanical properties of silica aerogels reinforced with nanofibers and microfibers [143].

Designing regenerable adsorbents that can also be reused over multiple cycles can minimize operational costs and lead to ecological sustainability. Nevertheless, repeated regeneration cycles can degrade the materials and lead to secondary contamination. Usually, aerogels can be regenerated using thermal or solvent extraction techniques. Thus, effective strategies must be developed to improve regeneration efficiency in removing harmful pollutants from wastewater [57,111,144,145]. Moreover, the effectiveness of regeneration is also based on the properties of the adsorbents. For example, bacterial cellulose aerogel can be reused if a suitable elution solvent is used. Lin et al. [146] determined the recyclability of attapulgite/bacterial cellulose aerogels and the removal efficiency of Pb^2+^ from wastewater. A hydrochloric acid solution was used to desorb Pb^2+^ ions from the prepared aerogels. After 5 cycles of adsorption-desorption, the removal efficiency of Pb^2+^ was maintained above 85%. Similarly, Sharma et al. [147] investigated the reusability of GO-doped modified silica aerogel (GO-SA) for efficiently removing organic dyes from an aqueous solution. The GO-SA aerogel showed a removal efficiency of >85% after 5 cycles.

More investigations are necessary to develop new materials with better selectivity and adsorption performance for specific contaminants, particularly pollutants that exist in low concentrations. Incorporating different materials and biomolecules like enzymes and antibodies into the structure of aerogels, their properties can be improved by adding specific functional groups, changing the pore size and shape, and increasing specific surface area [148]. For instance, Atoufi and collaborators studied the ion selectivity of green ambient-dried aerogels toward Pb^2+^ in the presence of competing Ca^2+^ and Mg^2+^ ions. The results demonstrated remarkable selectivity, with a (ion distribution coefficient) *K*_d_ value much higher than calcium and magnesium [149].

Production methods used for aerogel fabrication are a real concern for environmental health. Conventional drying techniques are known for having a high environmental impact due to their high energy consumption and excessive solvent use. Researchers are now exploring different manufacturing methods with minimal environmental impact, including 3D printing and plasma technology [150,151,152]. Also, rapid supercritical drying reduces high temperatures and solvent use and is more environmentally friendly than other drying processes [153]. Moreover, recycling the solvent from a mixture reduces the environmental impact [154].

Furthermore, the development of “green” adsorbents derived from non-harmful raw materials, biomass, geopolymers, and agricultural residues should be considered as important as the production processes. Although the reusability of adsorbents can lead to ecological sustainability, the disposal of these adsorbents represents an environmental concern. However, using “green” adsorbents, they can be used as soil amendments, providing agricultural benefits [155].

## 5. Future Directions and Research Gaps

In recent years, aerogel-based materials have gained considerable attention in water treatment. Significant laboratory studies have been conducted over the years to show their potential in removing toxic pollutants from aqueous solutions [97,156,157,158]. Therefore, their applications in real water systems need to be explored. A recent study investigated the adsorption efficiency of cellulose aerogel beads (CGP1) in simulated real wastewater prepared by adding humic acid (usually found in industrial wastewater) into the Cu(II) solutions (pH 5.6). The results showed an increase in the adsorption capacity of CGP1 beads (in the presence of humic acid) when the concentration of Cu(II) was increased. These results suggest that CGP beads could be a promising adsorbent with applications in real wastewater treatments [159].

Moreover, 3D graphene-biopolymer aerogels were manufactured and used for removing heavy metals, organic dyes, and organic solvents. The 3D-printed aerogel was also used for dye removal in a flow-through filtration study. An aqueous methylene blue (MB) solution was used to pass through a bottle-cap filtration system. In total, 100% of MB was removed after only 100 mL of water passed through the filter. These findings demonstrate the applicability of 3D-printed aerogel as a water filtration system on a larger scale [160].

Fluoride is a dangerous water pollutant, and it is difficult to remove using conventional methods. Mruthunjayappa et al. [161] studied the removal efficiency of fluoride by the continuous flow method using a bionanocomposite-based aerogel membrane (BNC-AG-0.1). Real water samples collected from five different places were spiked with 5 ppm fluoride. When applied for real-water purification, fluoride concentration was less than the WHO permissible limit, making the water safe to drink. These laboratory studies provide valuable information about the performance and parameters of adsorbents. Thus, the studied adsorbents could be further used in real environmental conditions by conducting large-scale experiments. For example, field filtration experiments were carried out in New Jersey for AS(V) removal using five hybrid adsorbents based on iron. The groundwater treated by the filter system showed a decrease in the As(V) concentration, demonstrating that these adsorbents could be used as effective filtration systems for As(V) removal [162]. Although aerogel research is still developing, all these studies and research results show that these materials have potential for use in wastewater treatment on a commercial scale.

Many adsorbents, such as activated carbon, metal–organic frameworks (MOFs), and polymer membranes, have costly manufacturing processes and can deteriorate over time. Thus, innovative aerogel-based materials have recently become of interest to improve their performance in removing pollutants due to their extraordinary properties. Although aerogel adsorbents demonstrated great potential as adsorbents for removing organic and inorganic contaminants in wastewater, more research studies are required to overcome the challenges that limit their applications. Thus, green and sustainable synthesis methods should be proposed, with minimal production costs and environmental impact. Another key recommendation for future research is to use less complicated drying stages that can lead to full-scale application and commercialization of aerogels. For example, the economic life cycle of three types of silica-based aerogels synthesized using ambient pressure drying was analyzed by Garrido et al. [163]. The synthesis of each aerogel was economically evaluated in terms of energy consumption, raw materials cost, and production costs. The results showed that optimizing the cost of the process minimized the time and energy consumption, making this process suitable for laboratory-scale activities. The purchase of raw materials is the most important economic factor. But using the ambient pressure drying method, two of the obtained aerogels required 20 days to dry, while the HYB-A aerogel required only four days to dry, representing only 0.49% of its total cost. Thus, compared to currently commercialized aerogels, the production costs will decrease once an industrial scale is used.

Another significant challenge is developing aerogel-based adsorbents with regenerative capacities. For example, renewable energy sources, such as solar or wind power, are advanced regeneration methods that can effectively lower production costs and reduce the environmental impact. The stability and reusability of aerogels can also lead to lower production costs, making them more commercially viable. A special focus is directed towards improving mechanical and chemical stability. By reinforcing them with different nanocomposite materials, the aerogel’s structure can be significantly enhanced. Moreover, advanced aerogels with improved properties can be used for removing multiple contaminants at once without losing their effectiveness under different conditions and several adsorption-desorption cycles [57,111,164].

As mentioned above, aerogels can adapt to different environmental factors. Thus, researchers are very interested in developing stimuli-responsive aerogels for wastewater treatment applications. Lately, applications of artificial intelligence (AI) and machine learning (ML) have been of great interest for monitoring water quality and the efficiency of adsorption technology [165]. By combining aerogels with AI, the performance of adsorbents in water purification and the water quality could be monitored. Designing innovative adsorbents for pollutant removal implies the development of a legislative framework that can ensure their safety in real-life applications (to prevent any risks to human health and the environment). Until now, few studies have investigated aerogels’ toxicity; therefore, their possible hazard assessment needs to be addressed. To prove their safety, a series of risk assessments must be performed before considering them suitable for commercialization [166]. Although developing a regulatory framework is complex and time-consuming, continuous research can significantly accelerate the approval of these new adsorbents. Fund pilot projects, government initiatives, and field trials are crucial for aligning research efforts with policy goals [167].

## 6. Conclusions

In recent years, important progress has been made in developing innovative aerogel-based adsorbents for water decontamination. This review presents a diverse range of aerogel adsorbents, including silica, polymer, carbon-based aerogels, and hybrid and composite aerogels, highlighting their advantages and disadvantages in removing pollutants from water and wastewater. The aerogels showed notable effectiveness in different water treatment applications with excellent adsorption capacities for various contaminants. Some research studies have proved that aerogels possess excellent selectivity towards specific ions, can be easily regenerated, and can be used multiple times without losing their adsorption capacities. Their customizable surface area, adjustable pore structure, and good mechanical strength led to the high removal efficiency of pollutants, making them suitable for large-scale water treatment. However, despite these outstanding results, aerogel-based materials present several challenges, thus limiting their applications in the market. Key technical and economic issues, such as sustainable synthesis methods, low cost, low energy consumption, effective regeneration techniques, and stability of aerogels, are significant challenges that need to be addressed when using these materials in real water systems. Additionally, regulatory and policy frameworks need to support continuous research in this field, ensuring that these high-performance adsorbents are safe for the environment and human health. Another key factor involves developing multifunctional adsorbents that can effectively target multiple contaminants simultaneously without losing their effectiveness under different environmental conditions. Lately, optimizing the adsorption processes by using AI and ML has represented a promising solution for monitoring water quality and the efficiency of adsorption technology. Looking ahead, great interest has been given to developing sustainable techniques such as photocatalytic degradation (that complements the adsorption process, thus being more effective in removing hazardous pollutants), capacitive deionization (CDI), hybrid membrane processes (that demonstrated better performance), and complexation–ultrafiltration (CP–UF) (that could be a promising technique for heavy metal removal). In summary, although there are still challenges in developing innovative aerogel-based materials, these materials offer promising solutions for future water purification systems.

## Figures and Tables

**Table 1:** Applications of aerogels in water decontamination.

Type of Aerogel	Pollutant/Application	Adsorption Capacity	Ref.
Modified silica aerogel	Pb (II)	N/A	[68]
Amidoxime-functionalized silica aerogels	Pb (II) Cu (II)	598.05 mg⋅g^−1^ 534.10 mg⋅g^−1^	[69]
C8/threonine-modified mesoporous silica aerogel	Azophloxine dye methylene blue dye	152.43 mg⋅g^−1^ 274.30 mg⋅g^−1^	[70]
Carbon nanomaterials-MTMS-based silica aerogel	Toluene Xylene	170 mg⋅g^−1^ 200 mg⋅g^−1^	[31]
Hydrophilic silica aerogel	Crystal violet dye	137.17 mg⋅g^−1^	[71]
Polyvinyl alcohol (PVA)-assisted cellulose nanocrystals/SiO_2_	Ciprofloxacin	163.34 mg·g^−1^	[72]
Magnetic mesoporous Fe_3_C/carbon aerogel	As	56.2 mg·g^−1^	[76]
Reduced graphene oxide−TiO_2_/sodium alginate aerogel	Ibuprofen Sulfamethoxazole	N/A	[79]
Graphene oxide/polyethyleneimine aerogel	Hg	N/A	[80]
3D reduced graphene oxides/Mn_3_O_4_ aerogel	antimonite (Sb(III)) antimonate (Sb(V))	151.84 mg·g^−1^ 105.50 mg·g^−1^	[81]
Cellulose nanocrystalline/polyvinylamine (PVAm)/reduced graphene oxide aerogel	Diclofenac sodium	605.87 mg g^−1^	[84]
Chitosan/zeolite composite aerogel	Indigo carmine dye Methylene blue dye	221 mg g^−1^ 108 mg g^−1^	[85]
3D polyaniline/cellulose nanofiber aerogel	Acid Red G dye Methyl Blue dye	600.7 mg g^−1^ 1369.1 mg g^−1^	[43]
Polyurea-crosslinked Ca-alginate aerogel beads	Pb (II) Diesel Mineral Pump oils	29 mg g^−1^ 4.9 mL/g^−1^ 6.1 mL/g^−1^ 4.9 mL/g^−1^	[86]
GO-TiO_2_/sodium alginate and rGO-TiO_2_/sodium alginate aerogels	photocatalytic degradation of microcystin-LR	N/A	[90]
TiO_2_-sodium alginate composite aerogels	Oil-water separation	N/A	[91]
Copper ferrite/reduced graphene oxide aerogel	Rhodamine B dye	N/A	[92]
3D graphene/δ-MnO_2_ aerogels	Pb^2+^ Cd^2+^ Cu^2+^	643.62 mg g^−1^ 250.31 mg g^−1^ 228.46 mg g^−1^	[93]
3D magnetic bacterial cellulose nanofiber/GO aerogel (MBCNF/GOPA)	Malachite green dye	270.27 mg g^−1^	[95]
Magnetic carboxymethyl chitosan (Fe_3_O_4_@PDA/CMC) aerogel	Methylene blue dye Crystal violet dye Methyl orange dye Congo red dye	289.6 mg g^−1^ 275.2 mg g^−1^ 82.07 mg g^−1^ 92.35 mg g^−1^	[96]
Magnetic carbon aerogel (MCA) containing Fe_3_O_4_ nanoparticles and sodium alginate (SA)	Cd (II)	143.88 mg g^−1^	[67]

## Data Availability

Not applicable.
